# Lovastatin Delays Infection and Increases Survival Rates in AG129 Mice Infected with Dengue Virus Serotype 2

**DOI:** 10.1371/journal.pone.0087412

**Published:** 2014-02-21

**Authors:** Marlen Martinez-Gutierrez, Luis A. Correa-Londoño, Jaime E. Castellanos, Juan C. Gallego-Gómez, Jorge E. Osorio

**Affiliations:** 1 Programa de Estudio y Control de Enfermedades Tropicales, Universidad de Antioquia, Medellín, Colombia; 2 Facultad de Medicina, Universidad de Antioquia, Medellín, Colombia; 3 Laboratorio de Patología, Congregación Mariana, Medellin, Colombia; 4 Grupo de Virología, Universidad El Bosque, Bogotá, Colombia; 5 Molecular and Translational Medicine Group, Viral Vector Core and Gene Therapy of the Neuroscience’s Group of Antioquia, Universidad de Antioquia, Medellín, Colombia; 6 Molecular and Translational Medicine Group, Universidad de Antioquia, Medellín, Colombia; 7 Department of Pathobiological Sciences, School of Veterinary Medicine, University of Wisconsin, Madison, Wisconsin, United States; CEA, France

## Abstract

**Background:**

It has been reported that treatment of DENV-infected cultures with Lovastatin (LOV), can affect viral assembly. The objective of this study was to evaluate the effect of LOV on the survival rate and viremia levels of DENV-2-infected AG129 mice.

**Methodology/Principal Findings:**

Mice were inoculated with 1×10^6^ plaque-forming units (PFU/ml) of DENV-2 and treated with LOV (200 mg/kg/day). Pre-treatment with one or three doses of LOV increased the survival rate compared to untreated mice (7.3 and 7.1 days, respectively, compared to 4.8 days). Viremia levels also decreased by 21.8% compared to untreated mice, but only in the group administered three doses prior to inoculation. When LOV was administered after viral inoculation, the survival rate increased (7.3 days in the group treated at 24 hpi, 6.8 days in the group treated at 48 hpi and 6.5 days in the group treated with two doses) compared to the untreated group (4.8 days). Interestingly, the serum viral titer increased by 24.6% in mice treated at 48 hpi with a single dose of LOV and by 21.7% in mice treated with two doses (at 24 and 48 hpi) of LOV compared to untreated mice. Finally histopathological changes in the liver and spleen in infected and untreated mice included massive extramedullary erythropoiesis foci and inflammatory filtration, and these characteristics were decreased or absent in LOV-treated mice.

**Conclusions/Significance:**

Our results suggest that the effect of LOV on viremia depends on the timing of treatment and on the number of doses administered. We observed a significant increase in the survival rate in both schemes due to a delay in the progression of the disease. However, the results obtained in the post-treatment scheme must be handled carefully because this treatment scheme increases viremia and we do not know how this increase could affect disease progression in humans.

## Introduction

Dengue is an arthropod-transmitted disease that has great importance worldwide. An estimated 50 million cases of dengue occur annually, especially in tropical and subtropical areas. In 2011, there were more than one million cases of dengue in the Americas alone, almost 20,000 with hemorrhagic complications, and an overall mortality rate of 0.07% [1]. In the last 50 years, the incidence of the disease has increased 30-fold, and it has even expanded into new geographic zones. This increase is due in part to global climate change. For example, through 2010, the El Niño phenomenon has been associated with an increase in the number of cases of dengue reported in Central and South America, including Colombia [2].

Dengue is caused by the dengue virus (DENV, official acronym), which is a member of the *Flaviviridae* family, genus *Flavivirus*. The disease is transmitted by mosquitoes of the genus *Aedes* (Diptera: *Culicidae*, Subgenus *Stegomyia*), with *Aedes aegypti* being the main vector in urban areas [3]. Four serotypes of DENV (1–4) can cause the disease, which can develop with a wide range of symptoms. Some patients develop a mild, self-limiting illness, while others progress into a severe illness characterized mainly by plasma extravasation that can be hemorrhagic or non-hemorrhagic [4]. Several classifications of the disease have been proposed; however, the most recent classification proposed by the World Health Organization is based on severity levels (severe and non-severe dengue). In addition, the non-severe dengue group includes both patients with warning signs of developing severe dengue and those who do not show these signs [5].

Currently, there is no licensed vaccine to prevent the development of the disease, nor is there any specific therapy against DENV infection. For this reason, the search for antiviral drugs derived from natural products or already licensed drugs that could have prophylactic and/or therapeutic use is a research priority [6]. Although in recent years a large number of studies have evaluated drugs with antiviral activity [7], only some drugs, such as chloroquine [8], balapiravir [9] and celgosivir [10], have been tested in clinical trials, and none of these have been licensed for use in humans.

The antiviral potential of Lovastatin (LOV) on DENV infection in cell culture has recently been reported [11]. This effect was reportedly due in part to faulty viral assembly [12]. A randomized controlled trial to evaluate the effects of LOV on adult patients infected with DENV has been proposed [13]. LOV is a drug that inhibits the enzyme 3-hydroxy-3-methylglutaryl coenzyme A (HMG-CoA) reductase [14]; this inhibition results primarily in decreased cholesterol synthesis but also affects other cellular processes such as isoprenoid synthesis and glycosylation due to the inhibition of dolichol synthesis. However, statins can also have “pleiotropic” effects on other cellular activities that are not directly dependent on cholesterol inhibition [15].

*In vitro* evaluations of compounds with antiviral potential are very useful; however, pre-clinical assays using animal models are essential for identifying the most promising drugs that can be evaluated and used in the medium-term in infected patients. Several DENV-infected mouse models have been used to model pathogenesis and the immune response and also for medication development and vaccine studies [16]. Among these is the AG129 mouse model, which is deficient in receptors for interferon (IFN) types I and II (IFN-α/β and IFN–γ, respectively).

The AG129 mouse model was established in 1995 to evaluate the antiviral response against lymphocytic choriomeningitis virus and vaccinia virus [17]. Several years later, this model was used to evaluate the immune response that developed after the inoculation of vaccine candidates against DENV [18]. In the last decade, AG129 mice have turned out to be a useful tool to study dengue in diverse fields, facilitating research in viral pathogenesis and immunity against infection [19]. In addition, these mice have been helpful in the evaluation of vaccine candidates [20] and drugs with potential antiviral activity [21], [22], [23], [24], [25].

Considering the previously reported *in vitro* antiviral activity of LOV and the utility of the AG129 mouse model for studying the antiviral potential of certain compounds, our objective was to evaluate the effect of LOV on viremia and survival in AG129 mice infected with DENV-2.

## Materials and Methods

### DENV Production

The New Guinea strain DENV-2 (ATCC VR-1255) was kindly donated by Dr. Richard Kenney (Division of Vector-Borne Infectious Diseases, Centers for Disease Control and Prevention, Ft. Collins, CO). For its production, C6/36 cells from *Aedes albopictus* (ATCC CRL-1660) were grown in 5% fetal bovine serum (Hyclone) at 28°C. The virus was passaged 10 to 15 times in tissue culture and then titered. To obtain viral titers greater than 1×10^7^ PFU/ml, the supernatants were collected and centrifuged for 1 hr at 30,000 g. The pellet was then resuspended in RPMI medium and stored at −70°C until use. For inoculations, the virus was resuspended in 50 µl of phosphate buffered saline (PBS) to a viral concentration of 5×10^5^ PFU/ml or 1×10^6^ PFU/ml.

### Ethics Statement

All of our experiments that involved the use of animals were approved by the Animal Experiment Committee of the University of Wisconsin and were carried out by properly trained researchers. All procedures were carried out in accordance with institutional guidelines and the National Institutes of Health Guide for the Care and Use of Laboratory Animals.

### Inoculation of AG129 Mice

AG129 mice were obtained from B&K Universal. Adult mice (six to eight weeks) were kept under *ad libitum* feeding conditions with 12 hr cycles of light and darkness. A 50-µl viral suspension containing either 5×10^5^ or 1×10^6^ PFU/ml was inoculated intraperitoneally. Animals were monitored for 30 min after inoculation to check for possible secondary effects on behavior.

### LOV Treatment

Recently, it has been reported that the antiviral effect produced by LOV in cell culture varies depending on the experimental strategy used. In cultures treated with LOV before inoculation (pre-treatment), the amount of viral protein decreased significantly, whereas in cultures receiving LOV after inoculation (post-treatment), the amount of both protein and viral genome increased significantly [11]. These results led us to postulate that, depending on the timing of LOV administration, the drug could produce antiviral effects through different mechanisms. For this reason, in the AG129 mouse model, we decided to evaluate two treatment schemes: pre- and post-inoculation treatment. Moreover, taking into account previous reports of antiviral activity in the AG129 mouse model [21], [22], the antiviral efficacy of LOV was defined in terms of a decrease in viremia or an increase in survival.

**Figure 1:**
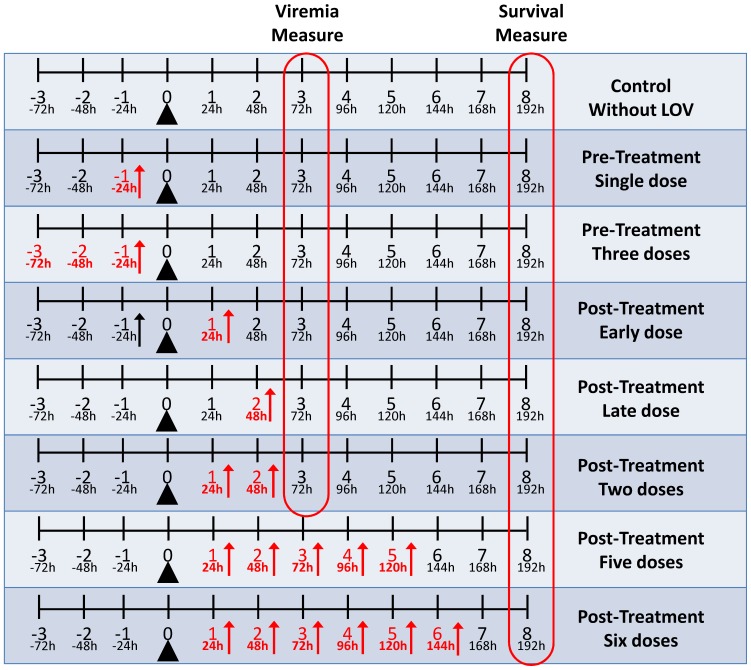
Mice treatment schemes. Groups of six AG129 mice were subjected to seven treatment schemes. The effect of LOV on viremia and survival rate before viral inoculation (pre-treatment) was evaluated in two experimental groups: single dose (only one dose 24 hours before viral inoculation) and three doses (72, 48 and 24 hours before viral inoculation). The effect of LOV on viremia and survival rate after viral inoculation (post-treatment) was evaluated in three experimental groups: Early dose (only one dose 24 after viral inoculation), late dose (only one dose 48 after viral inoculation) and two doses (24 and 48 hours after viral inoculation). Finally, two other groups were used to evaluate the effect of multiple LOV doses, administered only post-inoculation, on the survival rate (groups five and six doses). In the first five groups, at three days post-inoculation, blood samples were collected and viremia was quantified by a plaque formation assay in VERO cells. The black triangles indicate the day of viral inoculation, the red arrows indicate the days when the clinical signs were monitored, and the red lines indicate the experimental groups in which viremia or survival was measured. In all groups, animals were monitored twice a day and clinical signs (body weight, hackle hair, diarrhea, lethargy and paralysis) and survival rates were evaluated until day eight days, when the animals were sacrificed by CO_2_ asphyxiation.

Groups of six AG129 mice (six to eight weeks old) were subjected to seven different treatment schemes: Pre-treatment with single or three doses and post-treatment with early, late and two doses or five and six doses) (Figure 1). Moreover, in each assay we used three different types of controls groups: The “PBS” group consisted of mice that were not treated with LOV and were not infected but administered with PBS alone, the “CONTROL” group consisted of mice that were treated with LOV but were not infected and the “CONTROL-WITHOUT-LOV” group consisted of mice that were not treated with LOV but were inoculated with DENV-2. In both experimental strategies (pre and post-treatment), the viremia levels, loss of body weight and the survival rate of the CONTROL WITHOUT LOV group was compared with the treated groups. Finally, the CONTROL group was used to guaranty that the dose of LOV was not toxic.

In the treated groups, 200 mg/kg/day of LOV (Merck) was prepared in 50 µl PBS and administered orally. This dose was established by considering previous reports on the antiviral effect of LOV in mice infected with respiratory syncytial virus [26]. In that study, the authors tested three different doses of LOV: 0.5, 1 and 5 mg/day. Taking into account the average weight of a mouse (approximately 25 g), those doses correspond to human doses of 20, 40 and 200 mg/kg/day, respectively. We chose to use the 200 mg/kg/day dose (equivalent to 5 mg/day). The animals were monitored daily, and clinical signs (body weight, hackle hair, diarrhea, lethargy and paralysis) and the survival rate were evaluated until the completion of the study (eight days). Animals that showed clinical signs of severe illness prior to ending the study were sacrificed immediately by CO_2_ asphyxiation.

### Viremia Quantification from Blood Samples

At three and five days post-inoculation, blood samples were collected (approximately 100 µl) from immobilized animals by saphenous vein puncture. Serum samples were obtained by centrifugation after the blood coagulated. Viremia was quantified by a plaque formation assay in VERO cells. For this assay, 5×10^4^ cells were seeded per well in a 24-well plate, and triplicate serial dilutions of each sample (10^−1^–10^−5^) were prepared in DMEM and added to the cells. The serum was removed 1 hr after inoculation, and semisolid media was added (DMEM with 1.5% carboxymethylcellulose and 2% FBS). After seven days of incubation, the plates were stained with crystal violet and counted to obtain the titer in PFU/ml.

### Tissue Collection for Histopathological Analysis

Several animals were sacrificed at eight days post-inoculation by CO_2_ asphyxiation. The skin of each animal was then cleaned with 70% antiseptic alcohol, and tissues (spleen and liver) were dissected using surgical instruments. After dissection, the tissues were fixed in 10% formaldehyde for 48 hrs, processed in paraffin and stained with hematoxylin and eosin.

### Clinical Follow-up

The mice were monitored twice a day, and clinical signs (body weight, hackle hair, diarrhea, lethargy and paralysis) were recorded. The duration of the study was eight days (endpoint), but animals that showed clinical signs of severe illness prior to the end of the study were sacrificed immediately by CO_2_ asphyxiation, in accordance with the guidelines of the Animal Experimentation Committee. The survival rate of each group was recorded daily until the end of the study.

### Statistical Analysis

The viral titers in PFU/ml were tested for normality of distribution using a Kolmogorov–Smirnov test. When the titers had a normal distribution, an ANOVA-LSD test was used to compare levels of viremia between the treated and untreated groups. When the viral titers in PFU/ml did not have a normal distribution, we used a Kruskal-Wallis test to compare levels of viremia between treated and untreated groups. Kaplan-Meier survival curves were drawn and a Wilcoxon test was used to compare the survival between treated and untreated groups. Finally, to compare the average survival rates between treated and untreated groups, the ANOVA-LSD test was used. In all cases, p-values less than 0.05 were considered statistically significant.

## Results

### Comparison of Viremia Levels and Survival Rates of Mice Infected with Different Doses of the Virus

To determine the optimal dose for infection and the time of the highest levels of viremia, groups of six mice were infected intraperitoneally with either 5×10^5^ or 1×10^6^ PFU/ml of the New Guinea strain of DENV.

**Figure 2:**
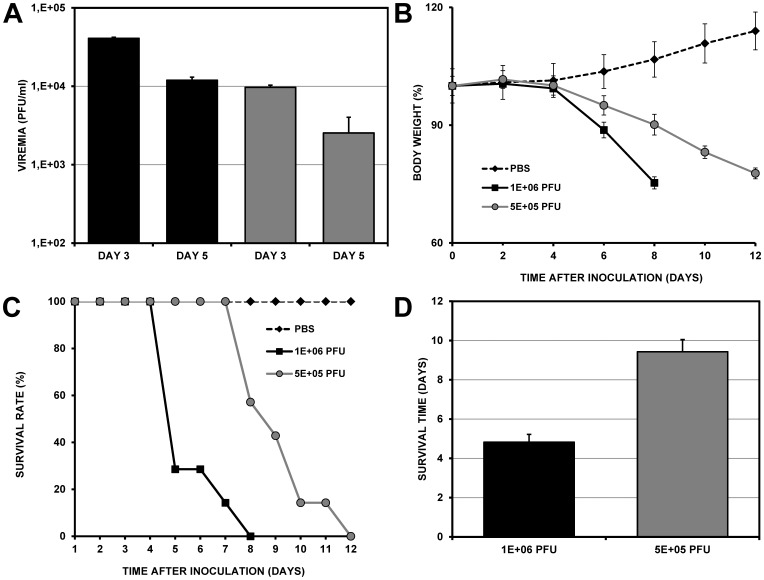
Effect of viral dose on viremia and survival rate of AG129 mice. Groups of six mice were inoculated intraperitoneally with a high viral dose of 1×10^6^ PFU/ml (dark bars) or a low viral dose of 1×10^5^ PFU/ml (grey bars) to determine the optimal dose of infection and time of occurrence of the highest levels of viremia. A. Plaque assay of infected VERO cells to quantify serum viremia at 3 and 5 dpi. Bars represent the standard error of the mean (SEM). B. Average body weight percentage, recorded every two days until the animals died or were sacrificed. C. Kaplan-Meier curve showing the survival rates of both groups from day 0 to day 12. D. Comparison of the survival rates of the two groups. Mice inoculated with 1×10^6^ PFU/ml developed higher levels of viremia at day 3.

At three and five days post-inoculation (dpi), serum samples were collected to quantify viremia by plaque assay in VERO cells. At 3 dpi, 4.1×10^4^ PFU/ml were detected when a high dose of the virus was inoculated, while half the dosage only produced a quarter of the viremia (1.2×10^4^ PFU/ml). Independently of the inoculated dose, there was a 70% decrease in viremia at 5 dpi (9.7×10^3^ with a high dosage of the virus and 2.5×10^3^ with half the dosage) (Figure 2A). Clinical signs and weight were recorded over twelve days. In the two infected groups, a considerable loss of weight was recorded compared to the control group inoculated with PBS. Weight loss began at 4 dpi and was maintained until days 8 (24.6%) and 12 (24.6%) in mice inoculated with the higher (1×10^6^ PFU/ml) and lower (5×10^5^ PFU/ml) doses of virus, respectively (Figure 2B). This weight loss was associated with the development of paralysis that prevented the mice from moving and getting food. In contrast, we found that the average survival (9.4±1.6 days) was significantly greater in the group inoculated with 5×10^5^ PFU/ml compared to mice inoculated with 1×10^6^ PFU/ml (4.8±1.6 days) (p<0.001) (Figure 2C–D). When animals showed clinical signs of severe paralysis with or without weight loss greater than 20%, they were sacrificed immediately by CO_2_ asphyxiation. The average survival rate was calculated based on the sum of days that each mouse survived (until death or sacrifice) over the number of mice in each experimental group. Because mice inoculated with 1×10^6^ PFU/ml developed higher levels of viremia, this dose was used for subsequent assays.

### Effect of Pre-treatment with LOV on Viremia and Survival Rate of DENV-2-infected Mice

**Figure 3:**
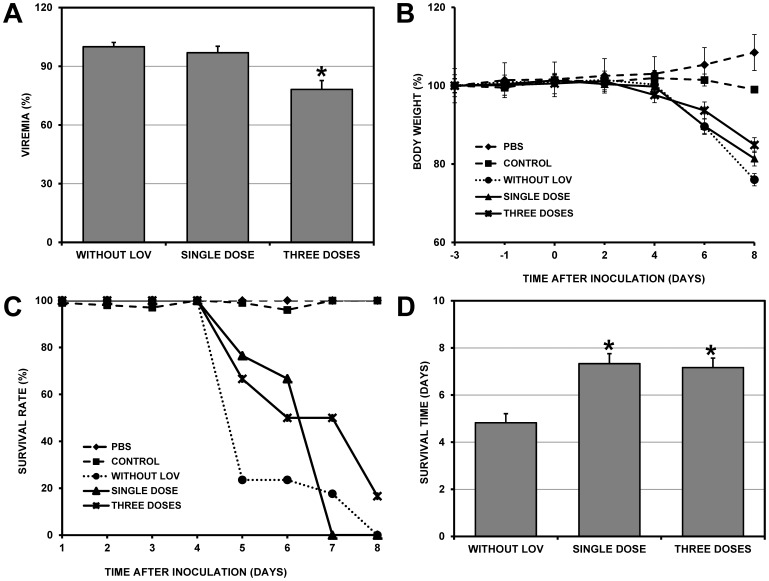
Effect of LOV treatment before viral inoculation (pre-treatment). Groups of six mice were treated with LOV (200 mg/kg/day) orally prior to intraperitoneal inoculation with 1×10^6^ PFU/ml of virus. One group was treated with a single dose (24 hrs before viral inoculation), and the other group was treated with three doses (72, 48 and 24 hrs before viral inoculation). Three types of controls groups were used. The “PBS” group consisted of mice that were not treated with LOV and were not infected, the “CONTROL” group consisted of mice that were treated with LOV but were not infected and the “CONTROL WITHOUT LOV” group consisted of mice that were not treated with LOV but were inoculated with DENV-2. The viremia and survival rates were compared between the control without LOV group and the treated groups (single- and three-dose groups). A. Plaque assay of infected VERO cells to quantify serum viremia at 3 dpi. Bars represent the standard error of the mean (SEM). B. Average body weight percentage, recorded every two days until the animals died or were sacrificed. C. Kaplan-Meier curve showing the survival rates of the untreated group and groups treated with one or three doses of LOV. D. Comparison of survival rates between the untreated group and groups treated with one or three doses. Asterisks in A and D indicate statistically significant differences between the treated LOV groups and the control without LOV group. In the group pre-treated with three LOV doses, there was a significant reduction in viremia. Moreover, the survival rates were increased in both groups (pre-treated with one or three doses).

Because recent reports have shown that the antiviral effect of LOV *in vitro* is dependent on the treatment scheme used (before or after inoculation), these same strategies were used in the *in vivo* AG129 mouse infection model. To determine the effect of LOV administered prior to viral inoculation (pre-treatment), groups of six mice were treated with a single dose (24 hrs) or three doses (72, 48 and 24 hrs) before inoculation with DENV-2 (Figure 1). Moreover, three different types of control groups were used (See Materials and Methods section). In the group pre-treated with three LOV doses, there was a significant 21.8% reduction in viremia compared to the control group without LOV (p<0.001) (Table 1). Viremia was not significantly reduced in mice treated with a single LOV dose (reduction of 3.0%) (Figure 3A). In the groups treated with one or three LOV doses, weight loss was reduced compared to the control group without LOV (18.6% and 15.1%, respectively, compared to 24.0%) (Figure 3B). Based on Kaplan-Meier analyses, we found a significant increase of survival in the groups treated with one or three doses compared to the control group without LOV (p<0.05). This result is consistent with the average survival rates, which significantly increased (p<0.001) in the groups treated with one or three doses compared to the control group without LOV (7.33±1.0 and 7.17±0.9 days, respectively, versus 4.8±1.6 days) (Figure 3C–D). The LOV treatment alone did not affect the weight or survival time of treated mice in the control group. However, despite the reductions in viremia and weight loss and the increased survival rate observed following LOV treatment, some animals in this experimental group (pre-treatment) developed severe paralysis and were sacrificed by CO_2_ asphyxiation the moment these signs started to develop.

**Table 1:** Viral titer in different treatment schemes compared with the control without LOV group.

TREATMENT SCHEME	NUMBER OF DOSES	VIRAL TITER (PFU/ml)	*p* VALUE
**PRE**	WITHOUT LOV	4.79E+04	–
	SINGLE DOSE	4.65E+04	0.447
	THREE DOSES	3.75E+04	0.000
**POST**	WITHOUT LOV	4.20E+04	–
	EARLY DOSE	4.30E+04	0.383
	LATE DOSE	5.20E+04	0.000
	TWO DOSES	5.20E+04	0.001

In all comparisons, *p*-values less than 0.05 were considered statistically significant.

### Effect of LOV Post-treatment on Viremia and Survival Rates of DENV-2-infected Mice

**Figure 4:**
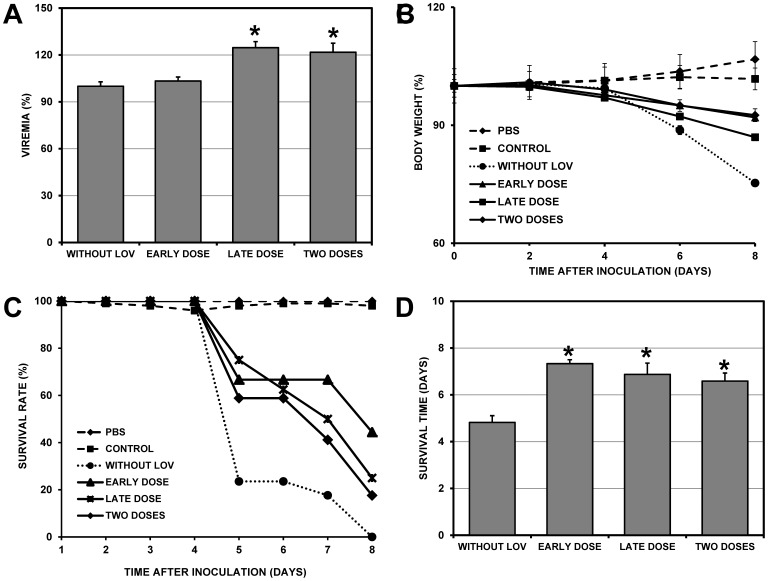
Effect of LOV treatment after viral inoculation (post-treatment). Groups of six mice were treated with LOV (200 mg/kg/day) orally with three treatment schemes: a single dose 24 hrs after inoculation (early dose), a single dose 48 hrs after inoculation (late dose) or two doses after inoculation (24 and 48 hrs). Inoculations were administered intraperitoneally with a viral dose of 1×10^6^ PFU/ml. Three types of control groups were used. The “PBS” group consisted of mice that were not treated with LOV and were not infected, the “CONTROL” group consisted of mice that were treated with LOV but were not infected and the “CONTROL WITHOUT LOV” group consisted of mice that were not treated with LOV but were inoculated with DENV-2. The viremia and survival rates were compared between the control without LOV group and the treated groups (early, late and two doses groups). A. Plaque assay of infected VERO cells to quantify serum viremia at 3 dpi. Bars represent the standard error of the mean (SEM). B. Average body weight percentage, recorded every two days until the animals died or were sacrificed. C. Kaplan-Meier curve showing the survival rates of the untreated group and the three treated groups. D. Comparison of survival rates between the untreated group and the three treated groups. Asterisks in A and D indicate statistically significant differences between the treated LOV groups and the control without LOV group. In the groups treated with a single dose at 48 h after viral inoculation or with two doses (24 and 48 hpi), there was a significant increase in viremia. Moreover, the survival rates were increased in the three groups.

We next evaluated the effect of LOV administered after viral inoculation (post-treatment). Groups of six mice were treated in three schemes: a single dose administered at 24 hours post-inoculation (hpi), a single dose administered at 48 hpi or two doses administered at 24 and 48 hpi. Moreover, three different types of controls groups were used (see the Materials and Methods section). The viral titer from mice treated at 24 hpi (4.3×10^4^ PFU/ml) was not different from the titer of mice without LOV (4.2×10^4^ PFU/ml). Surprisingly, in groups treated with a single dose at 48 hpi or with two doses (24 and 48 hpi), we found higher serum viral titers (5.2×10^4^ and 5.1×10^4^ PFU/ml, respectively) compared to the control group without LOV (4.2×10^4^ PFU/ml) (24.6% and 21.7% increases, respectively; p<0.001) (Figure 4A and Table 1). Weight loss in infected animals was 24%, while LOV treatment at 24, 48 and 24+48 hpi reduced weight loss to only 8.0%, 13.0% and 5.3%, respectively (Figure 4B). Using Kaplan-Meier analyses, we found a significant increase of survival in all three groups compared to the control group without LOV (p<0.05). The average survival rate of the untreated animals was 4.8±1.5 days. LOV treatment in the three post-treatment schemes significantly increased the survival rate to 7.3±0.5 days in the group administered a single dose at 24 hpi and 6.8±1.3 days in the group administered a single dose at 48 hpi. The survival rate in the group administered two doses was 6.5±1.4 days (Figure 4C–D). LOV treatment alone did not affect weight or survival time in the treated mice, as is indicated by the results observed in the CONTROL group. In these animal groups, some individuals developed severe paralysis and had to be sacrificed.

### Effect of Multiple LOV Doses Administered Post-inoculation on the Survival Rate of DENV-2-infected Mice

**Figure 5:**
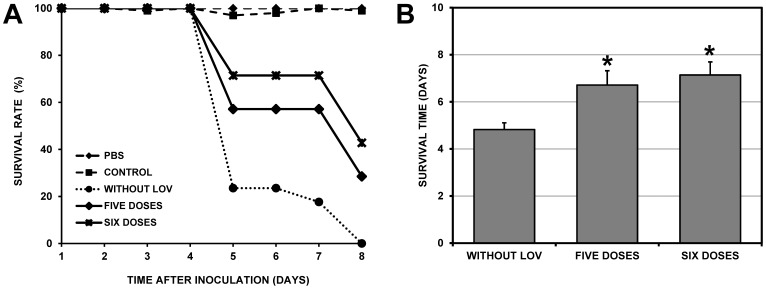
Effect of prolonged LOV treatment on the survival of infected mice. Groups of six mice were subjected to prolonged treatment with LOV (200 mg/kg/day) orally after viral inoculation (post-treatment). The first group received a daily dose for five days (24, 48, 72, 96 and 120 hrs) and the second group received a daily dose for six days (24, 48, 72, 96, 122 and 144 hrs). Inoculations were administered intraperitoneally with a viral dose of 1×10^6^ PFU/ml. Three types of controls groups were used. The “PBS” group consisted of mice that were not treated with LOV and were not infected, the “CONTROL” group consisted of mice that were treated with LOV but were not infected and the “CONTROL WITHOUT LOV” group consisted of mice that were not treated with LOV but were inoculated with DENV-2. The survival rates were compared between the control without LOV group and treated groups (five and six doses groups). A. Kaplan-Meier curve showing the survival rates of the untreated group and the two treated groups. B. Comparison of the survival rates of untreated and treated groups. Asterisks indicate statistically significant differences between treated LOV groups and the control without LOV group. Both treatment schemes, using multiple doses of LOV, significantly increased the survival rates.

To evaluate whether using multiple doses of LOV could increase the survival rate, groups of six mice were inoculated with DENV-2 (1×10^6^ PFU/ml) and treated with 200 mg/kg/day of LOV every 24 hrs for five or six days. Based on Kaplan-Meier analyses, we found a significant increase in survival in the groups treated with both treatment schemes compared to the control group without LOV (p<0.05). Specifically, the average survival rate was increased to 6.7±1.5 days in the five-dose group and 7.1±1.4 days in the six-dose group compared with the untreated control group (4.8±1.6) (Figure 5A–B).

### Histopathological Analysis

Spleens and livers of mice from each of the evaluated groups were dissected on day eight. Tissues were fixed and embedded in paraffin before being stained with hematoxylin and eosin. Tissue samples were examined at high resolution using an optical microscope. Below, we describe the observations for each experimental group.

#### Uninfected and untreated control mice

**Figure 6:**
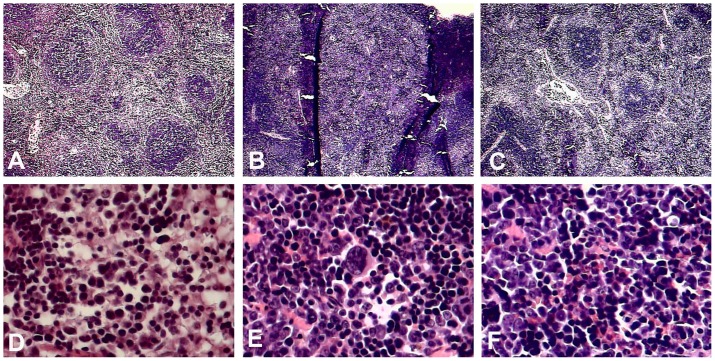
Histopathological analysis of spleen sections from AG129 mice under different experimental conditions. A, D. Untreated and uninfected control mice. B, E. Untreated infected mice. C, F. LOV-treated infected mice. A–C. Comparison between red and white pulp in spleen sections. In normal mice there was a conserved architecture of both pulps (A). In untreated infected mice, expansion of the red pulp was observed with depletion of the white pulp (B). In LOV-treated infected mice, a slight expansion of the red pulp was observed, but loss of the white pulp was not as severe as in untreated animals (C). D–F. Erythropoiesis comparison. In untreated infected mice, massive erythropoiesis was observed with megaloblastic changes (E). In normal mice (C) and treated mice (F), erythropoiesis was minor and no megaloblastic changes were observed.

**Figure 7:**
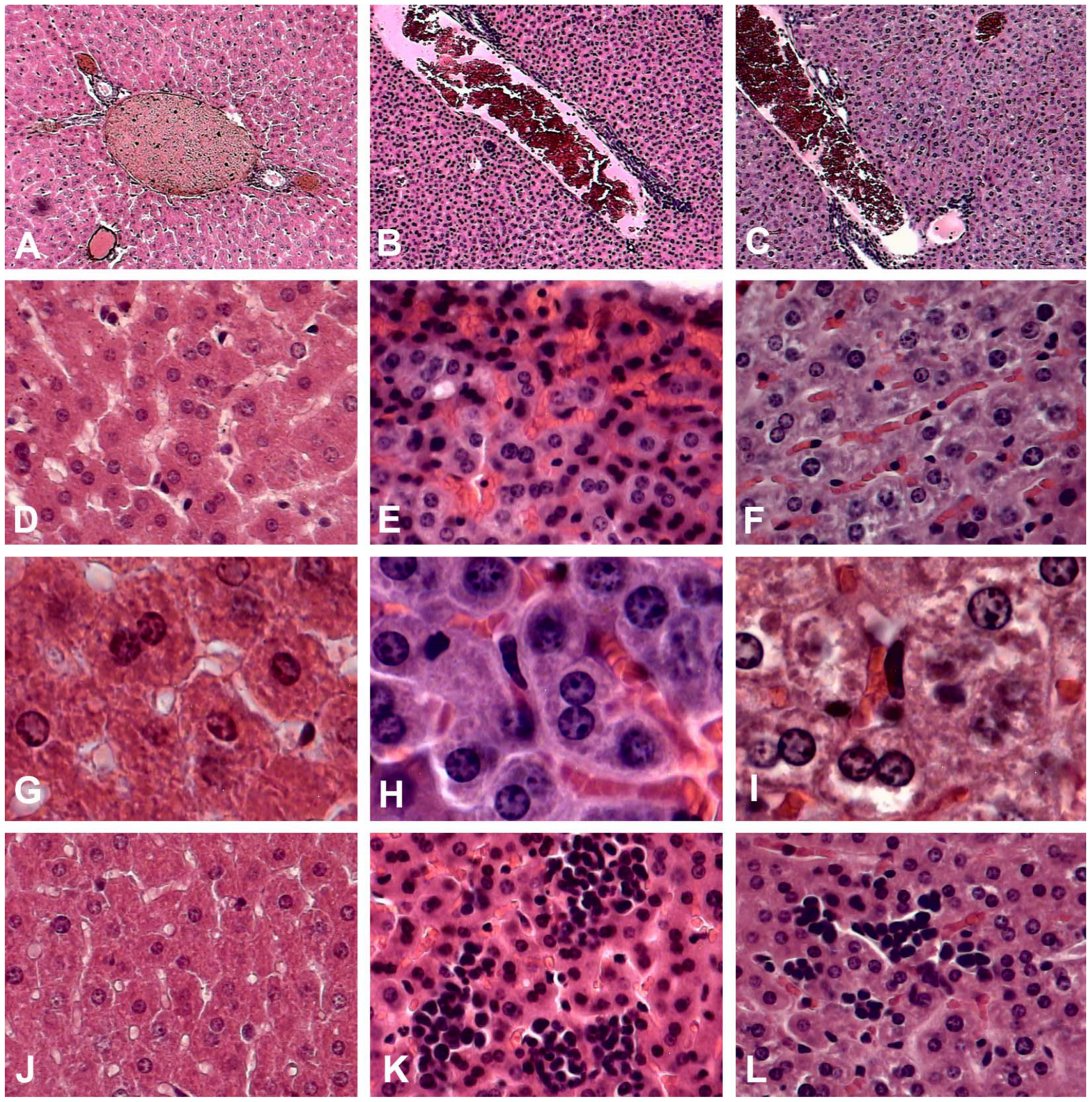
Histopathological analysis of liver sections from AG129 mice under different experimental conditions. A, D, G, J. Untreated and uninfected control mice. B, E, H, K. Untreated infected mice. C, F, I, L. LOV-treated infected mice. A–C. Comparison between venular portal sections. In infected mice (B) there was congestion with chronic inflammatory lymphocytes compared with normal mice (A), and in mice treated with LOV, the infiltration was lower (C). D–F. Sinusoids comparison. Sinusoidal congestion was observed in untreated infected mice (E), and this was reduced in LOV-treated mice (F). In normal mice, there was no congestion (D). G–I. In LOV-treated (I) or untreated (J) mice there was hyperplasia of Kupffer cells compared with normal mice (G). J–L. In infected mice, massive extramedullar erythropoiesis was observed (K). In LOV-treated mice, erythropoiesis was minor and the number of outbreaks was lower (L). In untreated and uninfected control mice, erythropoiesis was not observed (J).

In the spleen, there was a conserved architecture in the white and red pulp (Figure 6A), and moderate levels of erythropoiesis were present without megaloblastic changes (Figure 6D). The liver portal space lacked congestion or inflammatory infiltration (Figure 7A). The sinusoids did not present congestion (Figure 7D), and there was no hyperplasia in the Kupffer cells (Figure 7G), nor was there extramedullary erythropoiesis (Figure 7J).

#### Untreated infected mice

In the spleen, we observed red pulp expansion with white pulp reduction (Figure 6B) and extramedullary erythropoiesis with megaloblastic changes (Figure 6E). The liver showed congestion of the portal venule with the presence of a lymphocytic infiltrate (Figure 7B), sinusoidal congestion (Figure 7E), hyperplasia of Kupffer cells (Figure 7H) and abundant extramedullary erythropoiesis foci (Figure 7K).

#### Infected mice treated with LOV

In the spleen, a slight depletion of the white pulp (Figure 6C) and mild extramedullary erythropoiesis without megaloblastic changes (Figure 6F) were observed. The liver exhibited congestion of the portal venule with little lymphocytic infiltrate (Figure 7C), little sinusoid congestion (Figure 7F), hyperplasia of the Kupffer cells (Figure 7I) and scarce extramedullary erythropoiesis foci (Figure 7L).

## Discussion

Despite the large number of drugs that have been shown to have antiviral activity against DENV in *in vitro* models, few have been evaluated in animal models and only three drugs (chloroquine, balapiravir and celgosivir [8], [9], [10] have been tested in clinical assays in humans. One of the greatest difficulties in using antiviral drugs in humans is the licensing of new molecules, a process that can take many years. For this reason, *in vitro* and *in vivo* evaluation of licensed drugs that have been used and tested previously and have a low health risk (second-use drugs) is an interesting alternative in the search for new antivirals. Taking into account the advantages of evaluating second-use drugs, the present study demonstrates the effect of LOV on DENV infection in the AG129 mouse model.

To determine the optimal dose of infection and time of occurrence of the highest levels of viremia, preliminary experiments were performed in our lab to evaluate viremia from day 1 to day 9. We found that the viremia had a maximal peak at day 3, and at day 7 was almost absent (data not shown) as had been reported previously [18], [21]. We found that with a high viral dose, (1×10^6^ PFU/ml) viremia can reach titers on the order of 4.1×10^4^ PFU/ml, while titers reached with a low viral dose (5×10^5^ PFU/ml) were four times lower (1.2×10^4^) (Figure 2A). This dose-dependent effect has been reported previously in AG129 mice inoculated with the DENV-2 strain TSV01 [21] or with the DENV-2 strain D2Y98P [27]. With both viral doses (5×10^5^ or 1×10^6^ PFU/ml), the highest viremia level was measured at 3 dpi, but the viral load in the blood was significantly lower (greater than 70% reduction) at 5 dpi. These studies agree with previous results, in which inoculations with the DENV-2 strain TSV01 led to a peak in viremia levels at 3 dpi, followed by an 85% reduction in viremia at 5 dpi [21]. With the DENV-2 strain D2Y98P, the viremia peaked at 3 dpi with a reduction >90% at 5 dpi [27]. This reduction could be related to the activation of an immune response characterized by the appearance of IgM antibodies (beginning at 3 dpi) and neutralizing IgG antibodies (beginning at 4 dpi) [21]. Because mice inoculated with a high viral dose (1×10^6^ PFU/ml) developed higher levels of viremia and the viremia had its maximal peak at 3 dpi, that dose was used for subsequent assays and the viremia was evaluated only at 3 dpi. When comparing the survival of the two groups, we found that, in the group inoculated with the higher dose of virus, more than 70% of the mice had died by 5 dpi, and 100% of the mice were dead by 8 dpi (Figure 2C–D). A previous study using the same strain (New Guinea) reported 75% mortality at 8 dpi and 100% mortality at 14 dpi [22]. In addition, the infected animals lost increasing amounts of weight before death (Figure 2B). A possible explanation for this weight loss is the development of paralysis, which prevents the mice from moving around to obtain food. The development of paralysis has been described in AG129 mice infected with neuroadapted [18] and non-neuroadapted [28] strains of DENV inoculated intraperitoneally or intravenously and could be related to encephalitis development. Although encephalitis has also been reported in SCID mice inoculated intracerebrally with neuroadapted strains of DENV [29], neuronal infection by DENV is a controversial issue. Some authors have detected viral antigens and have isolated virus from nervous tissue [18] of mice inoculated by methods other than intracerebral inoculation, while others report a complete absence of viral antigens in the brain [30]. The neurotropism of DENV and the development of paralysis in DENV-infected mice are important observations. However, they make it difficult to use this strain for antiviral evaluations because when animals develop paralysis and lose more than 20% of their body weight, according to the regulations of the animal experimentation committee, they must be sacrificed immediately to avoid suffering.

In humans, it has been proposed that viral load is directly related to the severity of the disease and therefore, the survival of infected patients. Patients who develop severe dengue (with hemorrhage or hypovolemic shock) have higher titers than patients with milder forms of the disease [31], [32]. However, this relationship is controversial because no differences in viremia levels were found between adult patients with severe and non-severe forms of the disease in some studies [33]. Our results indicate that the progression of the disease depends on the initial viremia peak, being slower in mice with lower viremia at 3 dpi. These results agree with those of other studies, which reported that inoculation with a high viral dose led to animals dying a few days after the peak of viremia, whereas infection with a low viral dose led to asymptomatic dissemination with animals dying after the virus had been cleared [34]. However, regardless of the initial viremia levels, the mice end up developing paralysis, which is one of the disadvantages of the AG129 model. As we mentioned above, when animals develop paralysis they cannot move around to obtain food, therefore they lose weight (in some cases more that 20%) and according to the regulations of the animal experimentation committee, they must be sacrificed immediately to avoid suffering. This made it difficult, for example, to make antiviral evaluations in the long term.

Taking into account that the infection of AG129 mice with the New Guinea strain DENV-2 results in paralysis, it would be desirable to use a different strain that results in a viscerotropic infection type, such as the D2S10 strain DENV-2. This strain causes thrombocytopenia and vascular leakage in infected mice, characteristics more commonly associated with the infection in humans [35]. Recently, a mouse model has been developed that does not require immunocompromised AG129 mice. In this model, C57BL/6 mice are used, which are deficient in only the IFN-α/β receptor. The mice are infected with a different DENV-2 strain (D220) derived from the D2S10 strain. This D220 strain DENV-2 virus causes morbidity and mortality at a ten-fold lower dose than its parental strain, D2S10 [36]. For this reason, it would be a model more relevant for dengue studies (including antiviral evaluations). However, until now, this model has not been used for this type of study, and the AG129 model continues to be the most important model for evaluating possible antiviral candidates [16], [37].

In this study, we found only a single significant decrease of viremia (21.8%) (Figure 3A) among the groups that were treated with three doses of LOV prior to viral inoculation. LOV treatment is known to decrease the amount of cholesterol in the cell membrane [14], where it is an important component of lipid rafts [38]. These structures are important for DENV entry into its main cell targets, including monocytes/macrophages [39]. Taking into account the mechanisms of action described for statins, the antiviral effect of LOV could be related to its blocking of DENV cell entry, because the virus requires cholesterol-rich microdomains during its replication cycle [39] or because cholesterol is necessary for viral trafficking in late endosomes [40]. The antiviral effect could also be due to damage to isoprenylated proteins, which are required to complete the viral replication cycle once the virus enters the cell [41], [42]. Therefore, in mice treated with three consecutive doses of LOV, the drug might decrease the levels of cholesterol in the membrane, thus affecting the entry of the virus into the cell and leading to a decrease in viremia. It is possible that this effect might be related to the number of doses used, with one dose not sufficient to decrease cholesterol in the membrane. This scenario is consistent with the results of a previous study of rat hepatocytes, in which cholesterol inhibition was dependent not only on the LOV dose used but also on the duration of treatment [43].

Surprisingly, when animals were treated with two doses of LOV after viral inoculation (post-treatment experimental strategy), an increase in viremia was observed. It is known that LOV not only decreases cholesterol synthesis but also triggers pleiotropic effects that are independent of cholesterol inhibition [44]. For example, LOV inhibits the migration of inflammatory cells [45] by blocking the expression of leukocyte function antigen 1 (LFA-1), which binds to the cellular adhesion molecule ICAM-1 [46]. The formation of this LFA-1-ICAM-1 complex is responsible for the recruitment of leukocytes to sites of infection [47], [48]. As mentioned above, the correlation between viremia and disease progression/severity is not completely clear [31], [32], [33]. However, it has been demonstrated that the rapid removal of infected cells facilitates viral dissemination and thus increases the severity of the disease [49]. In contrast, mutant viruses that do not disseminate as easily to other tissues cause a transitory viremia increase that delays the progression of the disease [50]. It is likely that LOV could delay the migration of infected cells (monocytes and macrophages) to other tissues, thus inducing higher serum viral titers and delaying the progression to severe illness. However, when LOV treatment is removed, the infected cells can then migrate normally, leading to disease development. This hypothesis is in agreement with the histopathological analysis demonstrating that, in the livers of infected mice treated with LOV, there was a reduction in the infiltration of inflammatory cells, which were present in untreated infected mice (Figure 6A–C).

Despite our finding of increased survival rates in both treatment schemes (pre- or post-inoculation) as shown in the survival curves (Figure 3C–D, 4C–D and 5A–B), neither treatment was effective in preventing the death of the mice.

The post-treatment scheme is more similar to therapy in humans, because therapy would only be administered after infection. Thus, the results obtained here must be applied carefully because the post-inoculation treatment scheme increases viremia, and we do not know how this increase might affect disease progression in humans. As mentioned above, the relationship between viremia and disease severity is controversial. Therefore, additional studies are required to elucidate the mechanisms involved in the delay of dengue progression induced by LOV in spite of an increase in viremia.
